# Supplementary material for: Functional and structural insights into activation of TRPV2 by weak acids
Source: EMBO J. 2024 Apr 26;43(11):8. doi: 10.1038/s44318-024-00106-4 (PMC11148119; doi:10.1038/s44318-024-00106-4)
Supplement: Supplementary file 1 — Appendix [file 44318_2024_106_MOESM1_ESM.pdf]

Appendix

Functional and structural insights into activation of TRPV2 by weak acids

Ferdinand M. Haug[*1], Ruth A. Pumroy[*2], Akshay Sridhar[3], Sebastian Pantke[1], Florian Dimek[1], Tabea C. Fricke[1], Axel Hage[1], Christine Herzog[1], Frank G. Echtermeyer[1], Jeanne de la Roche[4], Adrian Koh[5], Abhay Kotecha[5], Rebecca J. Howard[6], Erik Lindahl[3,6], Vera Moiseenkova-Bell[#2], Andreas Leffler[#1].

[1]Department of Anesthesiology and Intensive Care Medicine, Hannover Medical School, 30625 Hannover, Germany. [2]Department of Systems Pharmacology and Translational Therapeutics, Perelman School of Medicine, University of Pennsylvania, Philadelphia, United States. [3]Department of Applied Physics, Science for Life Laboratory, KTH Royal Institute of Technology, Sweden. [4]Institute for Neurophysiology, Hannover Medical School, Hannover, Germany. [5]Thermo Fisher Scientific, Eindhoven, The Netherlands. [6]Department of Biochemistry and Biophysics, Science for Life Laboratory, Stockholm University, Sweden
*These authors contributed equally

#Corresponding authors:

Prof. Dr. med. Andreas Leffler
Department of Anesthesiology and Intensive Care Medicine
Hannover Medical School
Carl-Neuberg Strasse 1
30625 Hannover, Germany
Telephone: +49 511 532 8494
Email: leffler.andreas@mh-hannover.de

Prof. Vera Moiseenkova-Bell
Department of Systems Pharmacology and Translational Therapeutics
Perelman School of Medicine
University of Pennsylvania
Philadelphia, United States.
Telephone: +1 215 898-1154
Email: vmb@pennmedicine.upenn.edu

Table of contents:

Appendix Figures S1- S9
Appendix Tables S1 and S2

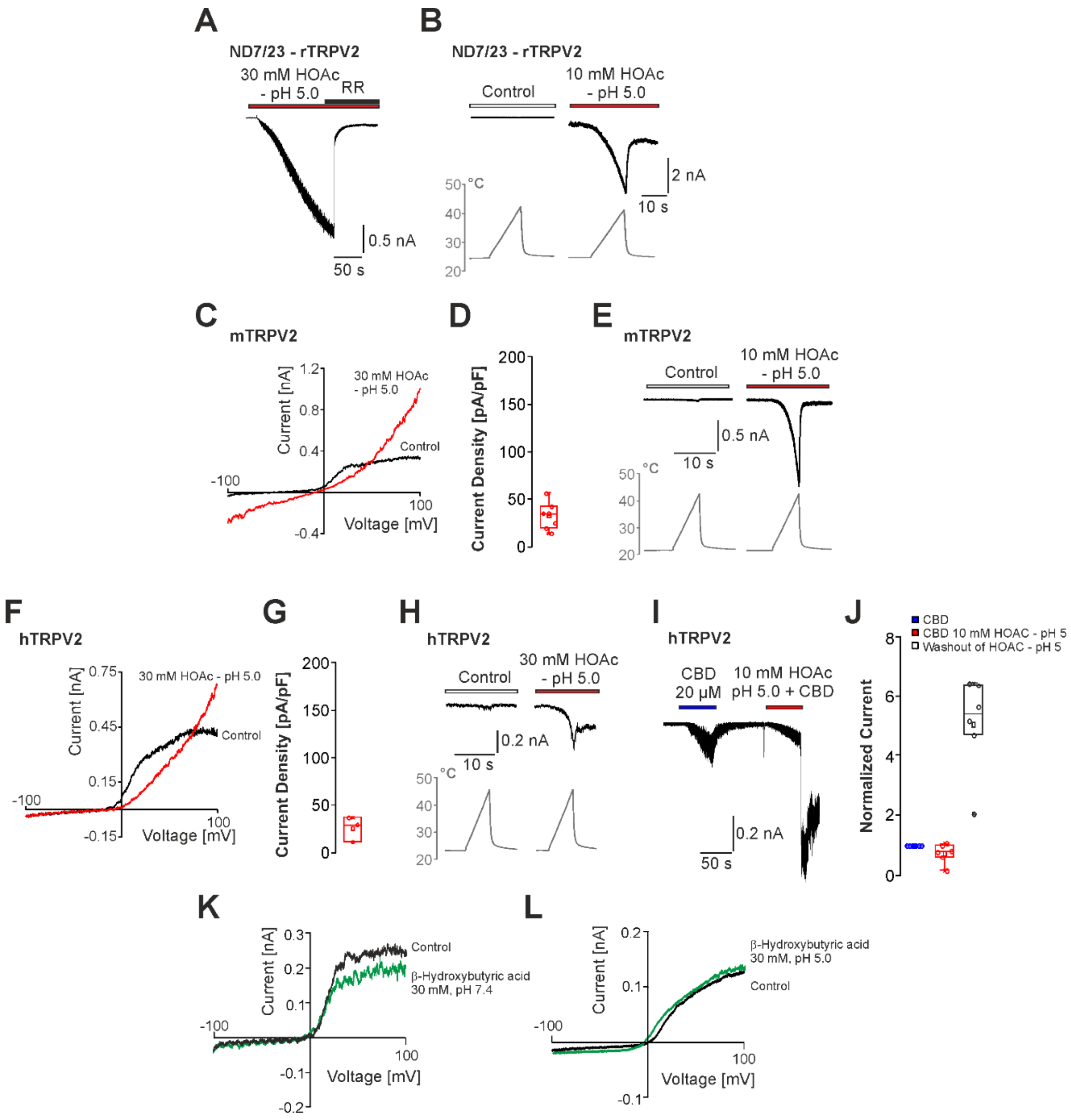

Appendix Figure S1. A and B. rTRPV2 expressed in neuroblastoma ND7/23 cells can be activated by 30 mM HOAc at pH 5.0 (A) and generates heat-evoked currents when exposed to 10 mM HOAc at pH 5.0 (B). C and F. Typical membrane currents observed in cells expressing mouse (m) TRPV2 (C) or human (h) TRPV2 (F) treated with 30 mM HOAc at pH 5.0. Depicted are also corresponding box diagrams with dot plots displaying the mean current densities at +100 mV (D and G). Currents were monitored during a 500 ms long voltage ramp ranging from -100 mV to +100 mV. E and H. Representative current traces from cells expressing mTRPV2 (E) or hTRPV2 (H) displaying heat-evoked inward currents by 10 or 30 mM HoAc at pH 5.0. I. Current traces on hTRPV2-expressing cells with two consecutive applications of 20 µM CBD. For the 2nd application, CBD was combined with 10 mM HOAC at pH 5.0. J. Box diagrams with dot plots displaying normalized current amplitudes of currents induced by CBD (set as 1), CBD + HOAc or following washout of HOAc. K and L. Typical membrane currents observed in cells expressing rTRPV2 treated with 30 mM β-hydroxybutyric acid at pH 7.4 (K) or pH 5.0 (L). Currents were monitored during a 500 ms long voltage ramp ranging from -100 mV to +100 mV.

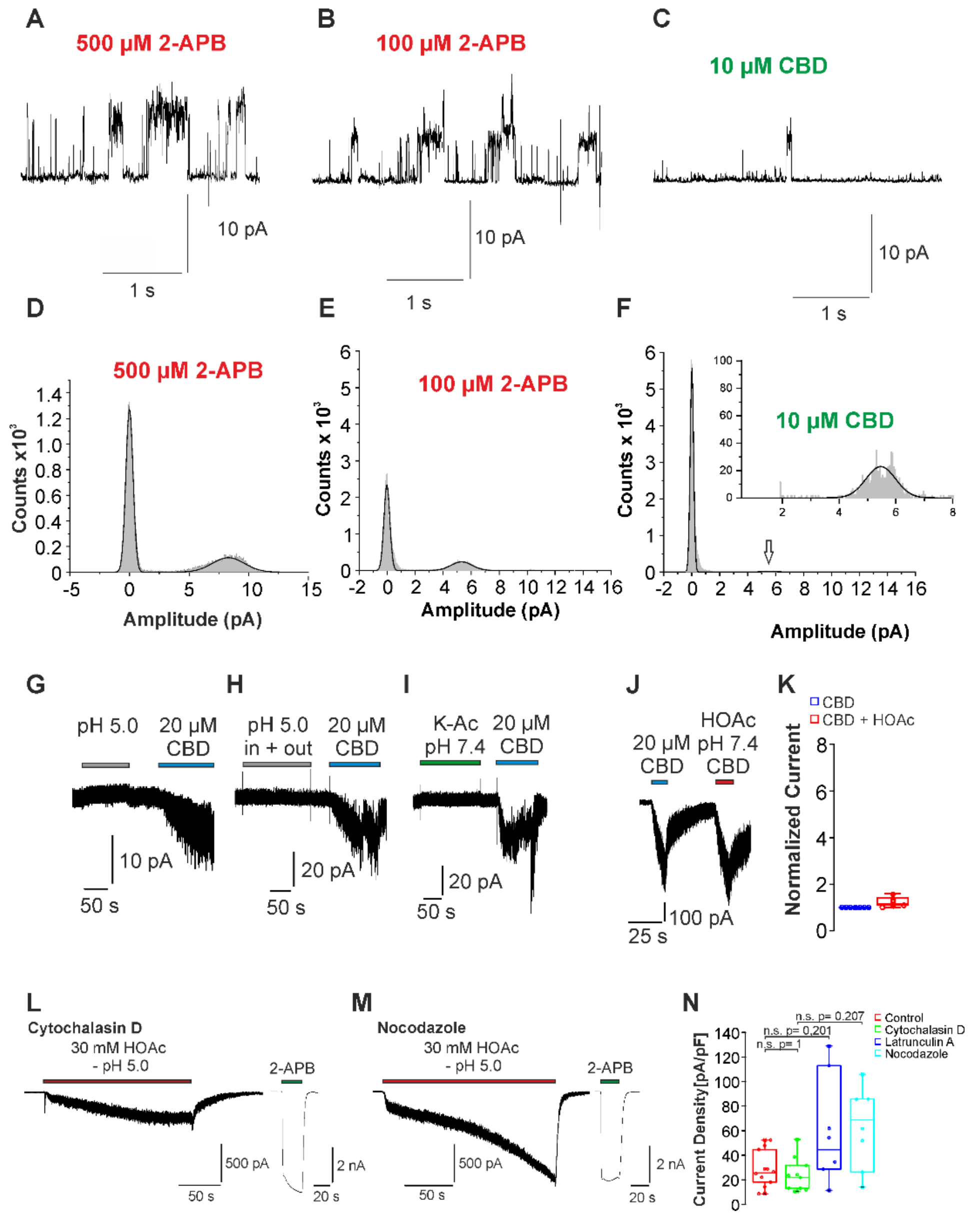

Appendix Figure S2.
A- C. Single-channel recordings with 500 µM 2-APB (A), 100 µM 2-APB (B) and 10 µM CBD (C) on rTRPV2 performed on inside-out patches held at +60 mV (n= 4 to 6 patches). D- F. Amplitude histograms for A- C. G. pH 5.0 titrated with HCl failed to induce channel openings in inside-out patches. H. The combined intracellular and extracellular acidification to pH 5.0 with HCl failed to activate membrane currents in inside-out patches. I. Application of 30 mM potassium-acetate (K-Ac) at pH 7.4 did not evoke membrane currents in patches containing rTRPV2. CBD was applied in order to verify expression of rTRPV2, experiments -60 mV (n= 5 each). J. Whole-cell recording demonstrating that 10 mM HOAc at pH 7.4 only induces a minimal potentiation of CBD-induced currents. K. Box diagrams with dot plots displaying the degree of potentiation induced by 10 mM HOAc at pH 7.4 on CBD-induced currents. L- M. Current traces evoked by 30 mM HOAC on pH 5.0 from HEK293T cells pre-treated with 10 µM cytochalasin-D (L) or 20 µM nocodazole (M). N. Box diagrams with dot plots displaying the current densities of HOAc-evoked inward currents in cells treated with 10 µM cytochalasin-D, 5 µM latrunculin or 20 µM nocodazole. n.s. denotes not significant. Comparison calculated with one-way ANOVA followed by Bonferroni correction (DF= 3, F-value= 3.75385).

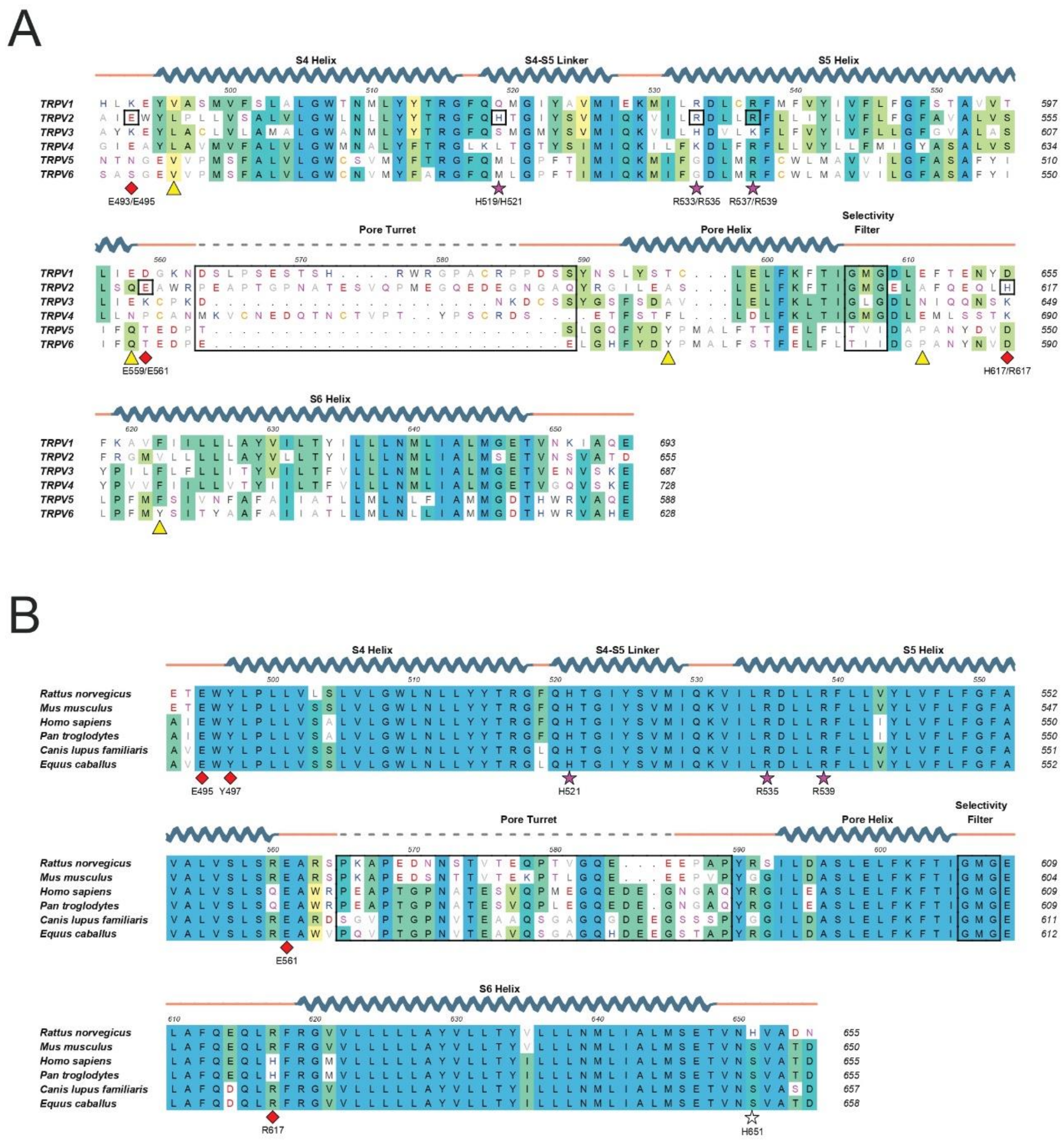

Appendix Figure S3. A, B. Sequence alignment of human TRPV channels, with the numbering at the top corresponding to human TRPV2 (A) and six species of mammalian TRPV2, with the numbering at the top corresponding to rat TRPV2 (B). Residues for the putative extracellular pH sensor are marked with a red diamond, those for the putative intracellular pH sensor are marked with a purple star. Residues identified as sensitive to protons in TRPV1 are marked with a yellow triangle. The pore turret and selectivity filter are marked by black boxes. Sequence conservation is indicated on a scale from yellow (least conserved) to blue (most conserved).

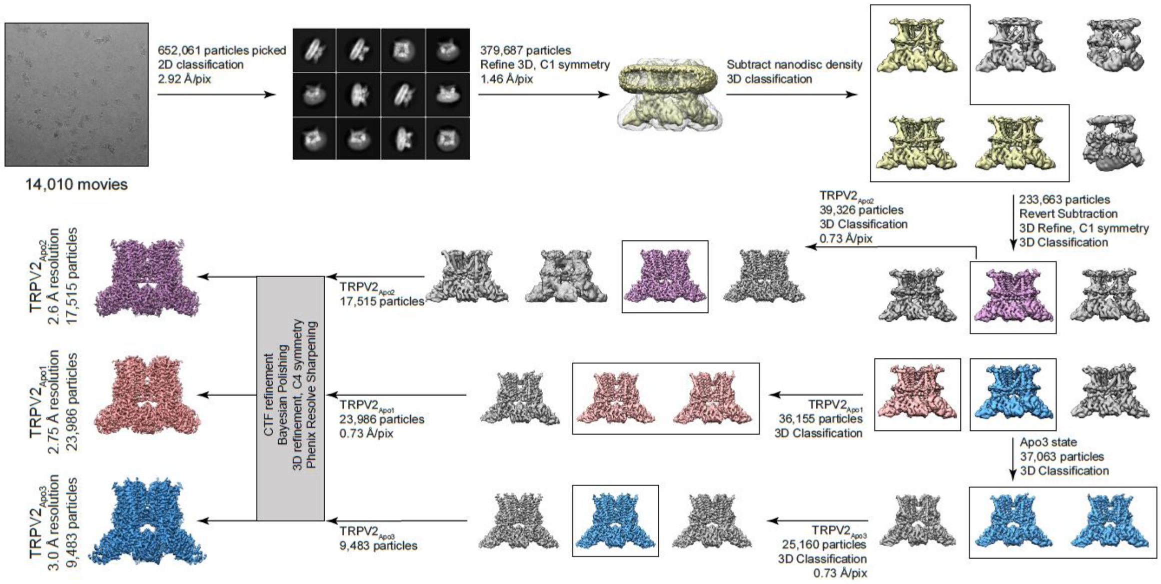

Appendix Figure S4. Processing tree for the apo TRPV2 dataset.

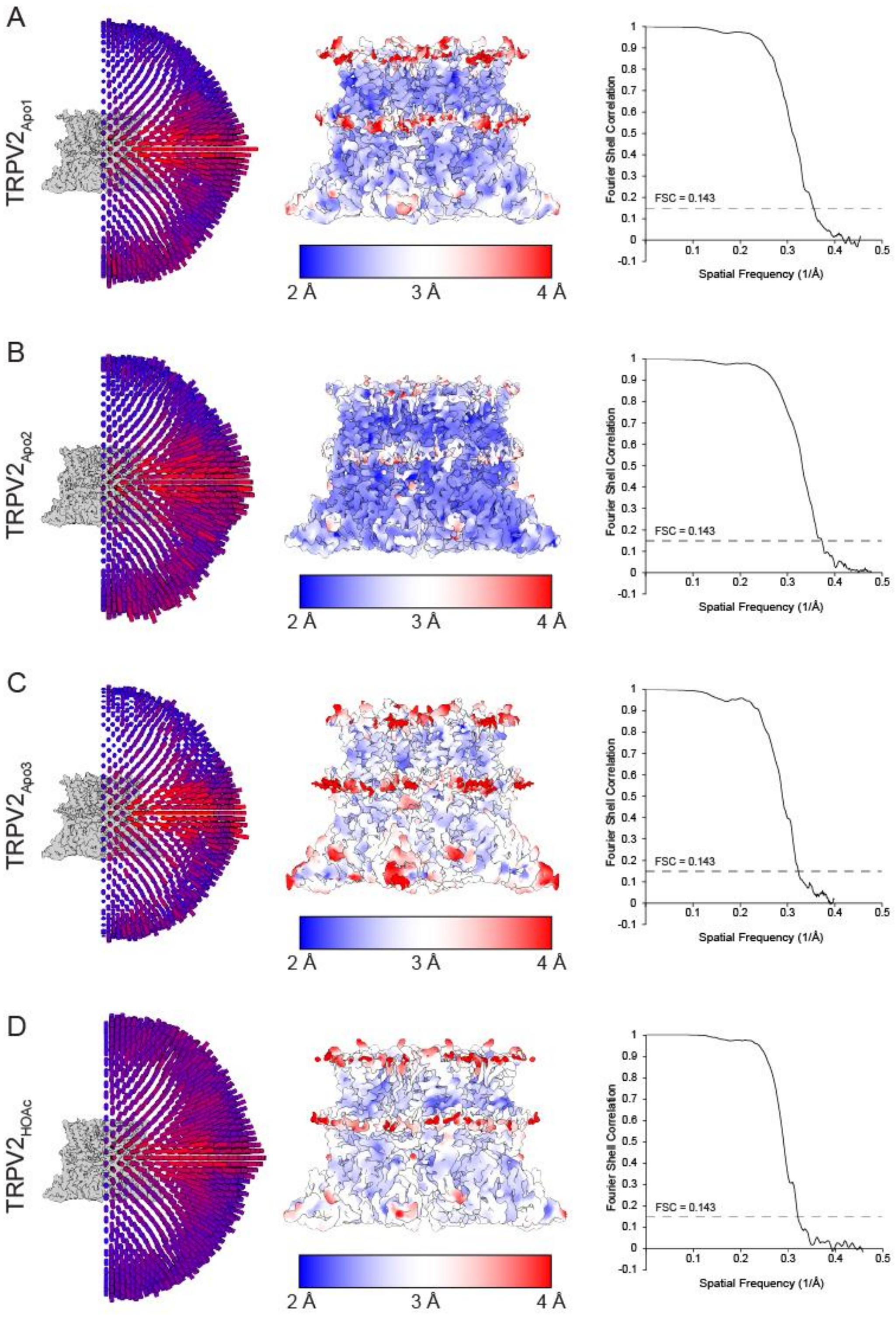

Appendix Figure S5. A- D. Data quality analysis of $TRPV2_{Apo1}$ (A), $TRPV2_{Apo2}$ (B), $TRPV2_{Apo3}$ (C), and $TRPV2_{HOAc}$ (D) showing angular distribution of particles (left panel), local resolution (middle panel), and the map FSC curve (right panel).

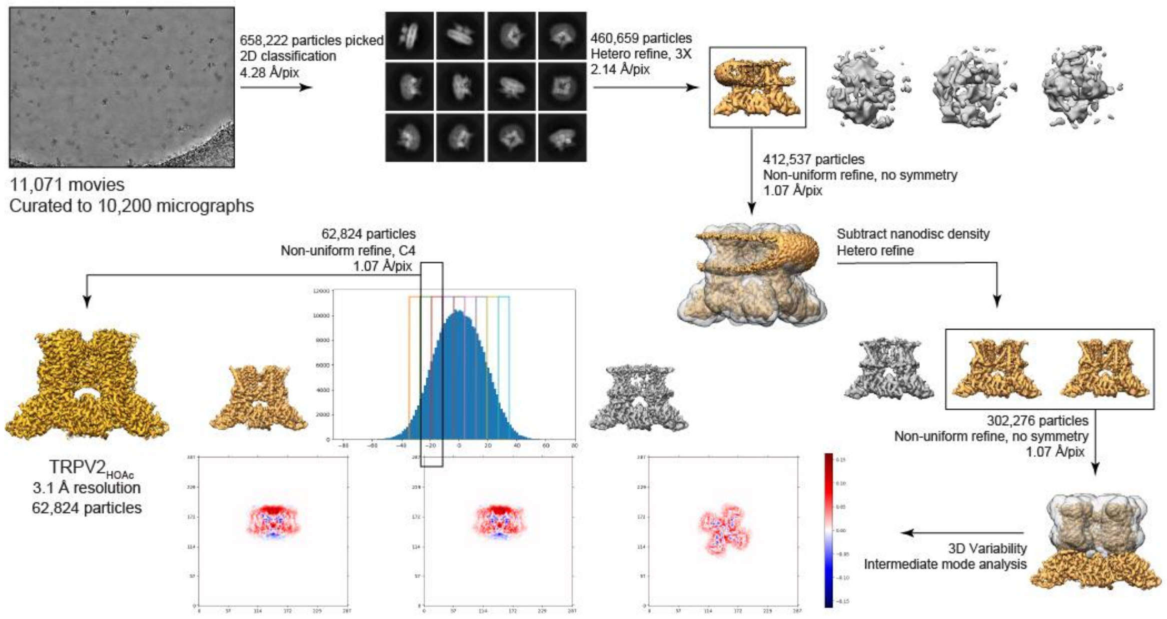

Appendix Figure S6. Processing tree for the weak acid TRPV2 dataset

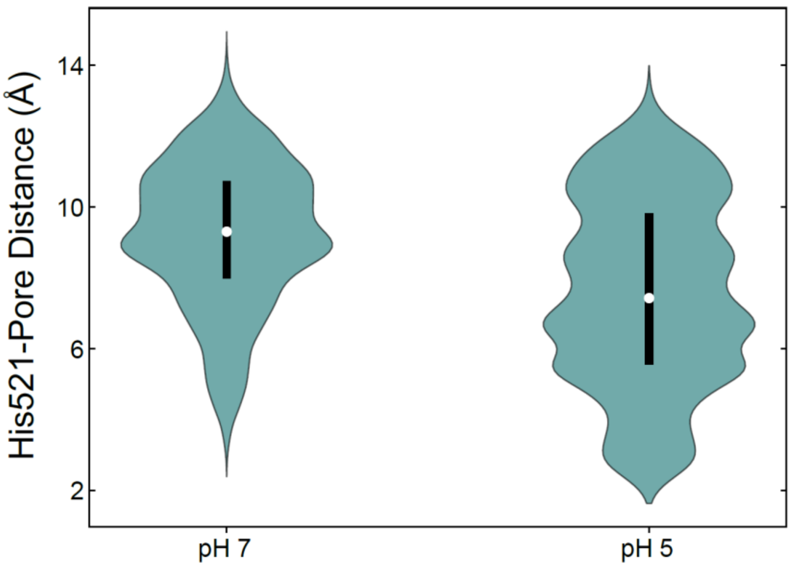

Appendix Figure S7. Negligible impact of the His651 residue on the channel's acid sensitivity. At both neutral and protonated states, the residue displays similar motility and orientation with regards to the central channel pore. Distances are calculated between the center-of-mass of each subunit's His651 residue and the center-of-mass of the C-alpha atoms of the four His651 residues. Violin plots contain data calculated from the final 120 ns of trajectory (n = 32000; 2000 frames each from 4 subunits and 4 simulation replicas). The median values are represented as white dots and the interquartile range is shown as a line.

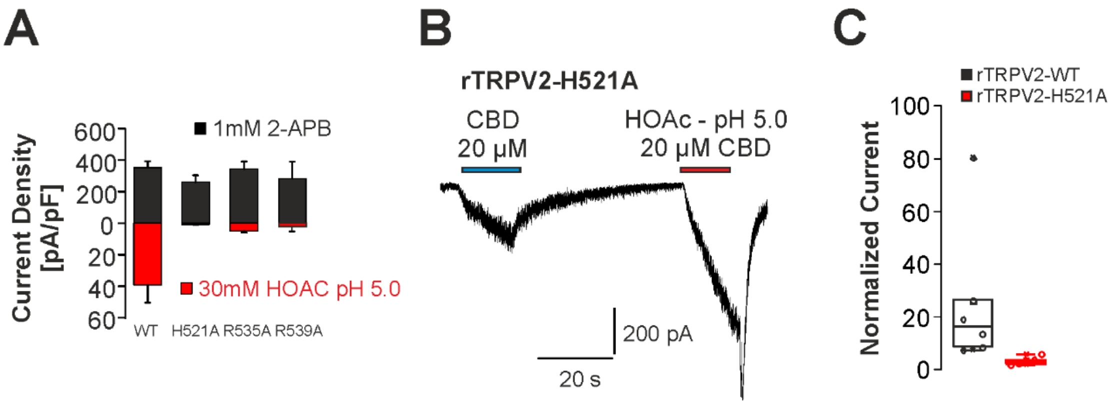

Appendix Figure S8. A. Bar columns displaying the current densities of heat-evoked currents provoked by 10 mM HOAc at pH 5.0 in cells expressing rTRPV2-WT, -H521A, R535A and and R539A. B. Typical current trace generated by rTRPV2-H521A. Currents were evoked by CBD or CBD in combination with 10 mM HOAc at pH 5.0. C. Box diagrams with dot plots displaying the degree of potentiation of CBD-induced currents evoked by 10 mM HOAc at pH 5.0 on rTRPV2-WT and rTRPV2-H521A.

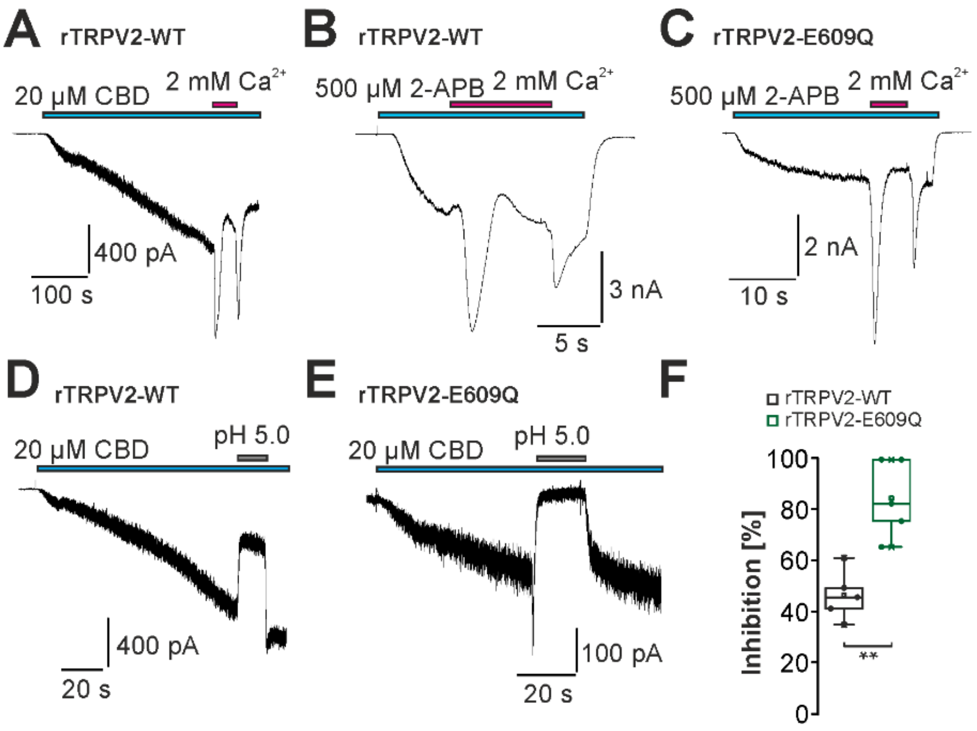

Appendix Figure S9. A and B. Typical current traces generated by rTRPV2-WT. Inward currents were induced by 20 µM CBD (A) or 500 µM 2-APB in nominal Ca^{2+}-free solution. A transient current increase for evoked by co-application with 2 mM Ca^{2+}. C. Typical current trace generated by rTRPV2-E609Q. The currents was induced by 20 µM CBD or 500 µM 2-APB in nominal Ca^{2+}-free solution. A transient current increase for evoked by co-application with 2 mM Ca^{2+}. D and E. Typical current traces generated by rTRPV2-WT and rTRPV2-E609Q. Inward currents were induced by 20 µM CBD, and a fractional block was induced by co-application of pH 5.0 titrated with HCl. F. Box diagram with dot plots displaying the degree of current inhibition induced in D and E by pH 5.0. ** denotes $p < 0.01$, unpaired t-test. Cells were held at -60 mV.

Appendix Table S1. Cryo-EM data collection and model statistics

	**TRPV2$_{Apo1}$** (EMD-28209, PDB 8EKP)	**TRPV2$_{Apo2}$** (EMD-28210, PDB 8EKQ)	**TRPV2$_{Apo3}$** (EMD-28211, PDB 8EKR)	**TRPV2$_{HOAc}$** (EMD-28212, PDB 8EKS)
**Data collection and processing**				
Magnification	165,000x	165,000x	165,000x	81,000x
Voltage (kV)	300	300	300	300
Camera	Falcon 4	Falcon 4	Falcon 4	K3
Defocus range (µm)	-0.5 to -2.0	-0.5 to -2.0	-0.5 to -2.0	-0.8 to -2.5
Pixel size (Å)	0.73	0.73	0.73	1.07
Micrographs	14,010	14,010	14,010	11,071
Particles from 2D classification (no.)	379,687	379,687	379,687	460,659
Final particles (no.)	23,986	17,515	9,483	62,824
Symmetry	C4	C4	C4	C4
Map resolution (Å)	2.75	2.6	3.0	3.1
FSC threshold	0.143	0.143	0.143	0.143
**Model Refinement**				
Model composition				
Nonhydrogen atoms	20,448	19,856	19,712	20,484
Protein residues	2,488	2,432	2,452	2,504
Ligands	8	8	4	4
R.M.S. deviations				
Bond lengths (Å)	0.004	0.004	0.004	0.003
Bond angles (°)	0.874	0.845	0.804	0.617
Validation				
MolProbity score	1.14	1.10	1.54	1.15
Clashscore	3.50	3.14	5.46	3.55
Rotamer outliers (%)	0.00	0.00	0.38	0.00
Ramachandran plot				
Favored (%)	98.69	98.65	96.35	98.05
Allowed (%)	1.31	1.35	3.65	1.95
Disallowed (%)	0.00	0.00	0.00	0.00

Appendix Table S2. pKa of ionizable groups within the Apo1 and HOAc structures calculated using PROPKA3.

Residue	Subunit	$TRPV2_{Apo1}$ pKa	$TRPV2_{HOAc}$ pKa
E495	A	4.19	4.89
	B	4.19	4.89
	C	4.19	4.89
	D	4.19	4.89
E561	A	5.13	5.66
	B	5.13	5.66
	C	5.13	5.66
	D	5.13	5.66
E599	A	5.2	6.32
	B	5.2	6.32
	C	5.2	6.32
	D	5.2	6.32
E609	A	4.71	5.14
	B	4.6	4.92
	C	4.71	4.75
	D	4.6	4.97
H521	A	5.49	6.25
	B	5.49	6.25
	C	5.49	6.25
	D	5.49	6.25
H651	A	5.2	6.67
	B	5.95	6.67
	C	5.33	6.67
	D	5.81	6.67
