# Supplementary material for: Functional and structural insights into activation of TRPV2 by weak acids
Source: EMBO J. 2024 Apr 26;43(11):8. doi: 10.1038/s44318-024-00106-4 (PMC11148119; doi:10.1038/s44318-024-00106-4)
Supplement: Supplementary file 10 — Expanded View Figures [file 44318_2024_106_MOESM10_ESM.pdf]

Expanded View Figures

Figure EV1. Weak acids inhibit endogenous PAC-mediated currents in HEK293T cells.

(**A**) BCECF-based ratiometric imaging on non-transfected HEK293T cells treated with pH 5.0 titrated with HCl, or 10 or 30 mM HOAc at pH 5.0. Application of HOAc, and to a lesser degree pH 5.0 induced a reversible decrease in fluorescence ratio, correlating with an intracellular acidosis. (**B**) Representative current trace from a non-transfected HEK293T cell demonstrating that 30 mM HOAc at pH 5.0 fails to evoke slowly activating currents without TRPV2 in the cell. Note the very rapidly inactivating currents emerging at the beginning of the recordings, demonstrating proton-evoked activation of endogenous acid-sensitive ion channels (ASIC1a) in some HEK293T cells. (**C**) Membrane currents in non-transfected HEK293T cells challenged with pH 5.0 or the combination of pH 5.0 and 100 μM pregnenolone sulfate. (**D**) Box diagrams with dot plots displaying normalized currents at +100 mV from cells described under C ($n = 5$). (**E**) Membrane currents in non-transfected HEK293T cells challenged with pH 5.0 or the combination of pH 5.0 ($n = 9$) and 10 ($n = 5$) or 30 mM ($n = 9$) HOAc. (**F**) Box diagrams with dot plots displaying normalized currents at +100 mV from cells described under (**E**). (**C**, **E**, **G**) Currents were monitored during a 500 ms long voltage ramp ranging from −100 mV to +100 mV. (**G**) Voltage-dependent membrane currents in a non-transfected HEK293T cell evoked by 200 ms long pulses from −60 to 120 mV applied in steps of 20 mV. Currents were evoked in control solution and in 10 mM HOAc at p 5.0. (**H**) Current–voltage plots of experiments performed in (**G**) ($n = 4$). Peak current amplitudes were normalized to the amplitude evoked at 120 mV. (**I**, **J**) Current traces on rTRPV2-expressing cells with two consecutive applications of 200 μM 2-APB. For the 2nd application, 2-APB was combined with pH 5.0 with HCl (**I**) or with 10 mM HOAC at pH 5.0 (**J**). (**K**) Box diagrams with dot plots displaying normalized current amplitudes of currents in induced by 2-APB (set as 1), 2-APB + pH 5.0 ($n = 8$) or 2-APB + HOAc ($n = 8$). (**D**, **F**, **K**) The box denotes the 50th percentile (median) as well as the 25th and 75th percentile. The whiskers mark the 5th and 95 percentiles. Data points beyond the whiskers are outliers. Source data are available online for this figure.

 © The Author(s)

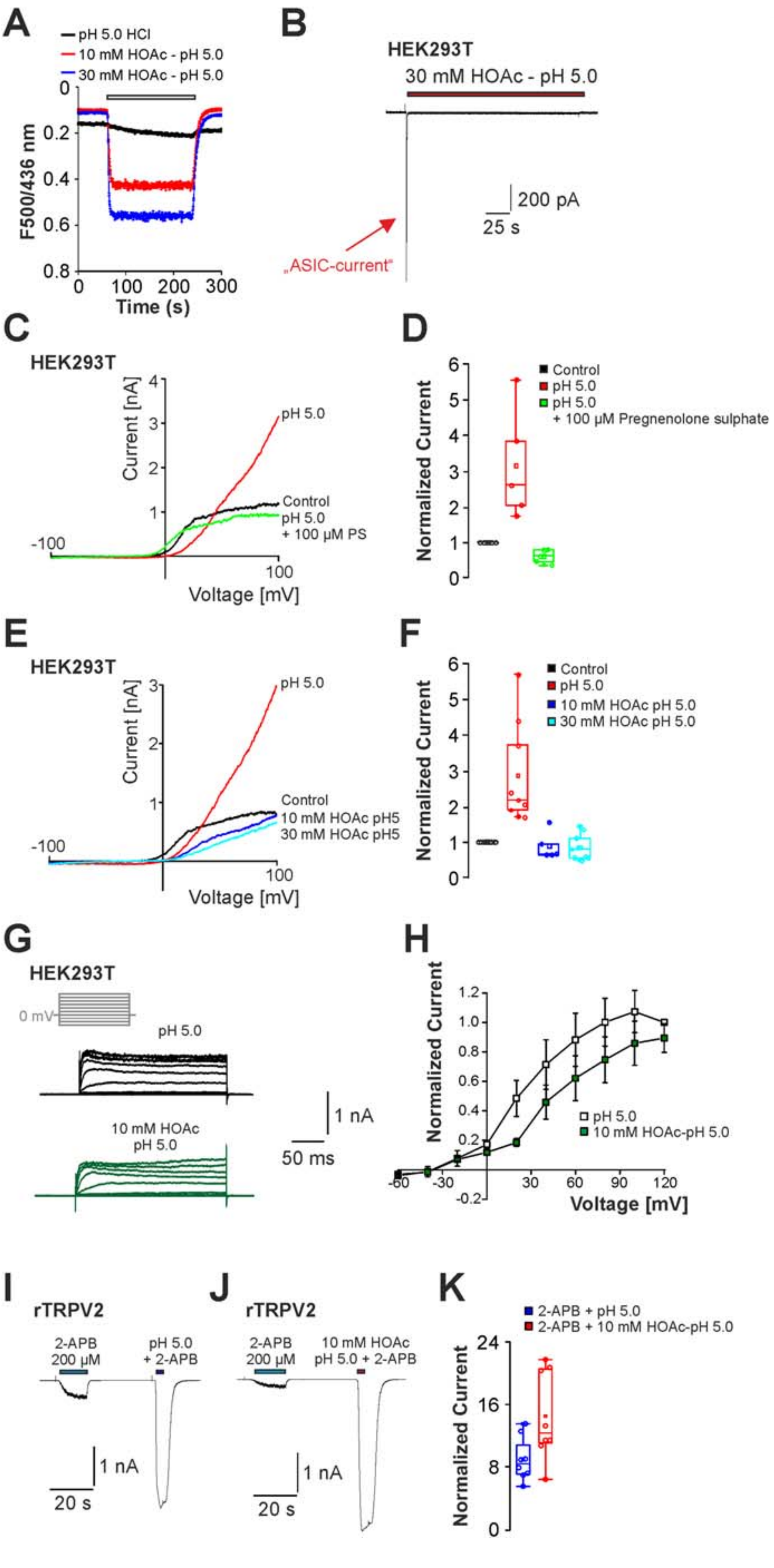
A
pH 5.0 HCl
10 mM HOAc - pH 5.0
30 mM HOAc - pH 5.0
F500/436 nm
Time (s)
B
HEK293T
30 mM HOAc - pH 5.0
200 pA
25 s
„ASIC-current"
C
HEK293T
Current [nA]
pH 5.0
Control
pH 5.0
+ 100 µM PS
Voltage [mV]
D
Normalized Current
Control
pH 5.0
pH 5.0
+ 100 µM Pregnenolone sulphate
E
HEK293T
Current [nA]
pH 5.0
Control
10 mM HOAc pH5
30 mM HOAc pH5
Voltage [mV]
F
Normalized Current
Control
pH 5.0
10 mM HOAc pH 5.0
30 mM HOAc pH 5.0
G
HEK293T
0 mV
pH 5.0
10 mM HOAc
pH 5.0
1 nA
50 ms
H
Normalized Current
pH 5.0
10 mM HOAc-pH 5.0
Voltage [mV]
I
rTRPV2
2-APB
200 µM
pH 5.0
+ 2-APB
1 nA
20 s
J
rTRPV2
2-APB
200 µM
10 mM HOAc
pH 5.0 + 2-APB
1 nA
20 s
K
2-APB + pH 5.0
2-APB + 10 mM HOAc-pH 5.0
Normalized Current

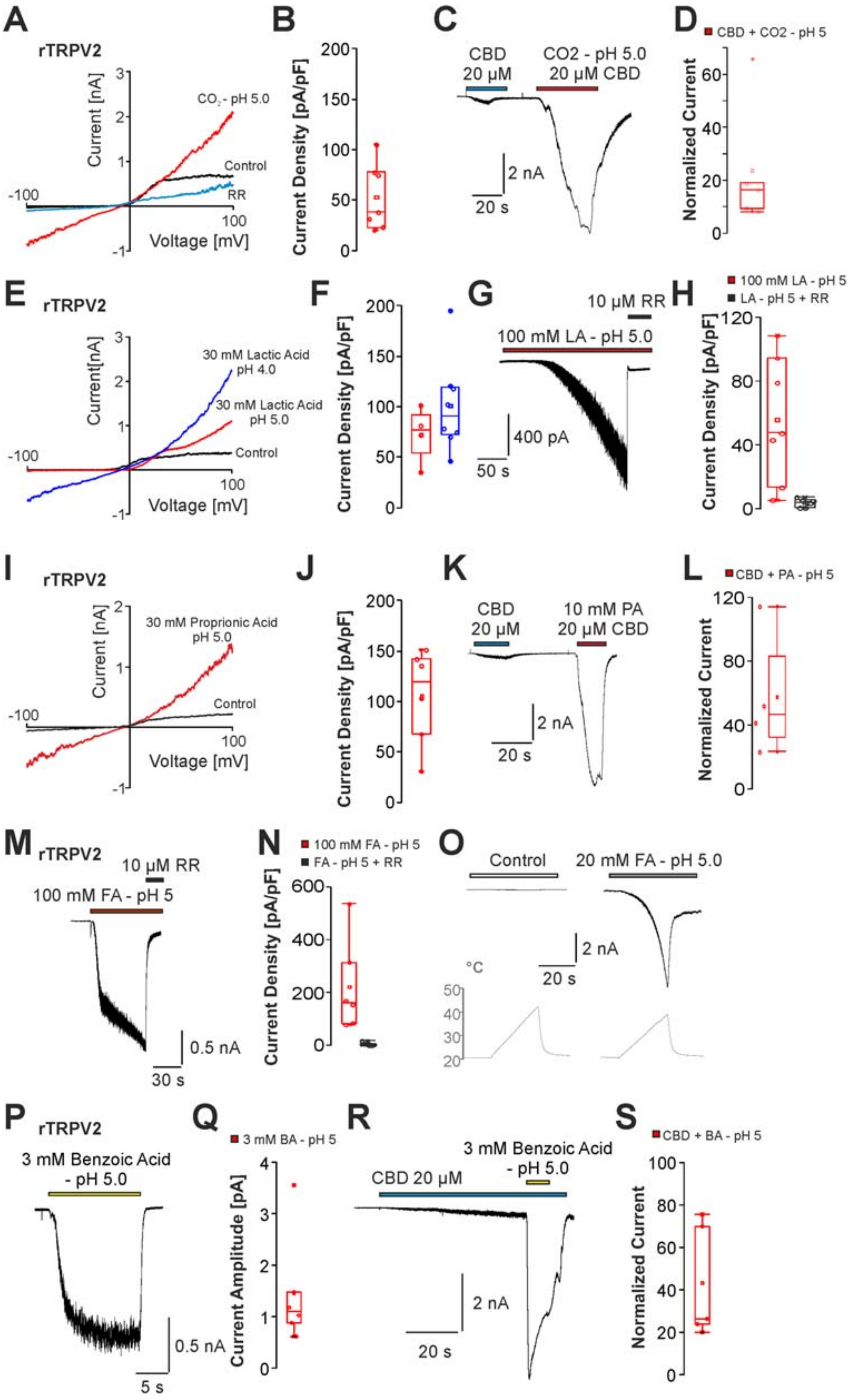
A
rTRPV2
CO2 - pH 5.0
Control
RR
Current [nA]
Voltage [mV]
B
Current Density [pA/pF]
C
CBD 20 µM
CO2 - pH 5.0 20 µM CBD
2 nA
20 s
D
CBD + CO2 - pH 5
Normalized Current
E
rTRPV2
30 mM Lactic Acid pH 4.0
30 mM Lactic Acid pH 5.0
Control
Current[nA]
Voltage [mV]
F
Current Density [pA/pF]
G
10 µM RR
100 mM LA - pH 5.0
400 pA
50 s
H
100 mM LA - pH 5
LA - pH 5 + RR
Current Density [pA/pF]
I
rTRPV2
30 mM Proprionic Acid pH 5.0
Control
Current [nA]
Voltage [mV]
J
Current Density [pA/pF]
K
CBD 20 µM
10 mM PA 20 µM CBD
2 nA
20 s
L
CBD + PA - pH 5
Normalized Current
M
rTRPV2
10 µM RR
100 mM FA - pH 5
0.5 nA
30 s
N
100 mM FA - pH 5
FA - pH 5 + RR
Current Density [pA/pF]
O
Control
20 mM FA - pH 5.0
2 nA
20 s
°C
P
rTRPV2
3 mM Benzoic Acid - pH 5.0
0.5 nA
5 s
Q
3 mM BA - pH 5
Current Amplitude [pA]
R
CBD 20 µM
3 mM Benzoic Acid - pH 5.0
2 nA
20 s
S
CBD + BA - pH 5
Normalized Current

Figure EV2. Several weak acids activate TRPV2.

(**A**) Membrane currents observed in cells expressing rTRPV2 challenged with CO_2 at pH 5.0. (**B**) Box diagrams with dot plots displaying the mean current densities at +100 mV induced by CO_2 ($n = 7$). (**C**) Current trace from a rTRPV2-expressing cell generating inward currents induced by CBD or CBD in combination with CO_2. (**D**) Box diagrams with dot plots displaying the degree of potentiation induced by CO_2 ($n = 5$) The CO_2-solution was achieved by bubbling the extracellular solution with CO_2 using a commercially available soda streamer. (**E**) Membrane currents induced by 30 mM lactic acid at pH 5.0 ($n = 4$) and 4.0 ($n = 8$). (**F**) Box diagrams with dot plots displaying the mean current densities at +100 mV induced by lactate. (**G**) Activation of an inward current in a rTRPV2-expressing cell exposed to 100 mM lactate at pH 5.0. The inward current was blocked by co-application with 10 μM ruthenium red (RR). (**H**) Box diagrams with dot plots displaying the current densities of lactate-induced inward currents in rTRPV2-expressing cells ($n = 7$). (**I**) Membrane currents induced by 30 mM propionic acid at pH 5.0 on rTRPV2. (**J**) Box diagram with dot plots displaying the mean current densities at +100 mV induced by propionic acid ($n = 6$). (**K**) Current trace from a rTRPV2-expressing cell generating inward currents induced by CBD or CBD in combination with propionic acid at pH 5.0. (**L**) Box diagram with dot plots displaying the degree of potentiation induced by propionic acid. (**M**) Inward current induced by 100 mM formic acid (FA) at pH 5.0 on rTRPV2 ($n = 4$). The inward current was blocked by 10 μM ruthenium red (RR). (**N**) Box diagrams with dot plots displaying the current densities of formic acid-induced inward currents in rTRPV2-expressing cells ($n = 6$). (**O**) Representative traces displaying heat-evoked currents induced by 20 mM formic acid at pH 5.0 on a rTRPV2-expressing cell. (**P**) Inward current induced by 3 mM bezoic acid at pH 5.0 on rTRPV2. (**Q**) Box diagrams with dot plots displaying the current densities of benzoic acid-induced inward currents ($n = 6$). (**R**) Original trace from a rTRPV2-expressing cell generating inward currents induced by CBD or CBD in combination with 3 mM benzoic acid at pH 5.0. (**S**) Box diagram with dot plots displaying the degree of potentiation induced by benzoic acid ($n = 5$). In (**B**, **D**, **F**, **H**, **J**, **L**, **N**, **Q**, **S**), the box denotes the 50th percentile (median) as well as the 25th and 75th percentile. The whiskers mark the 5th and 95 percentiles. Data points beyond the whiskers are outliers. Source data are available online for this figure.

© The Author(s)

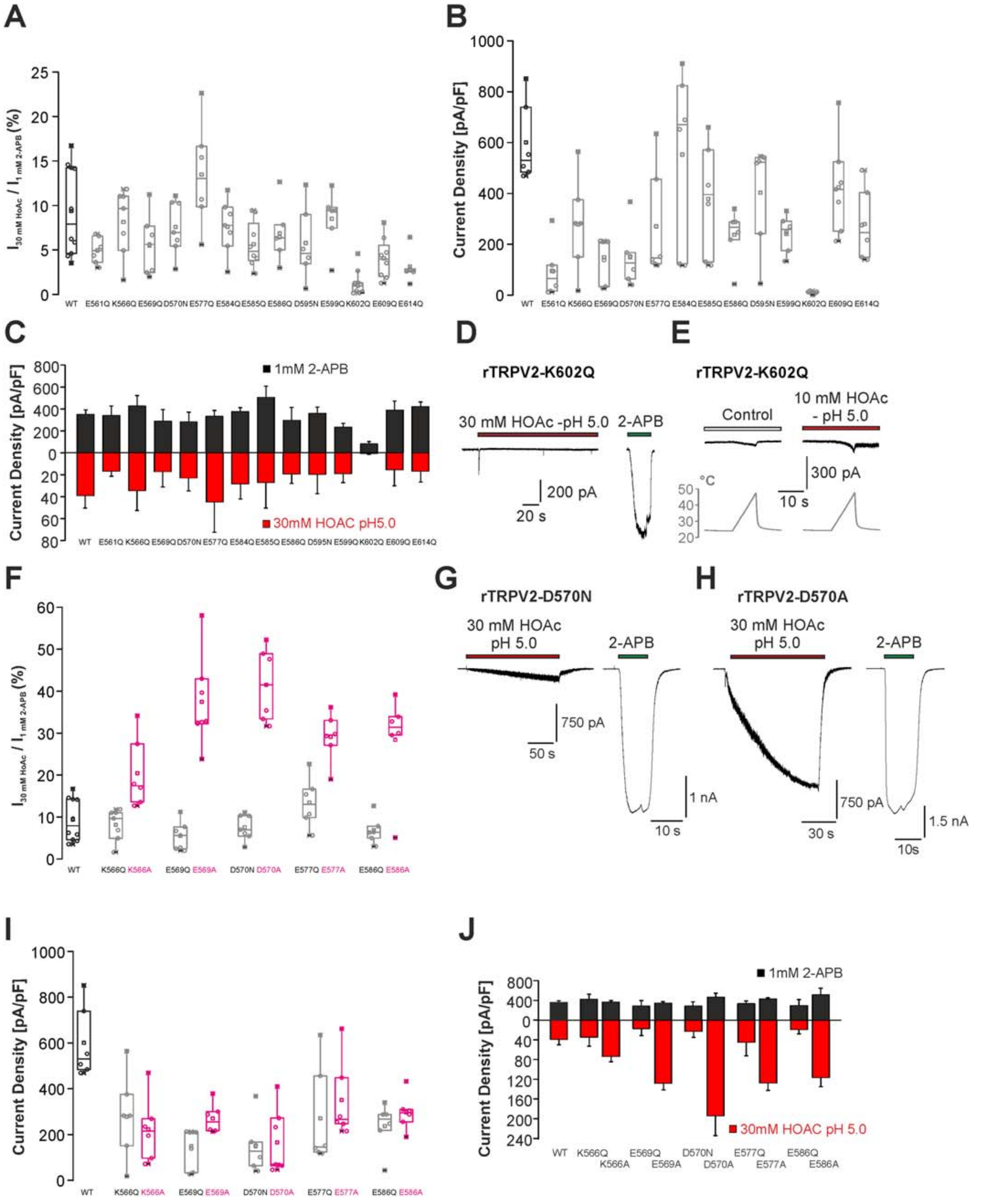
A
B
C
D
E
F
G
H
I
J
rTRPV2-K602Q
30 mM HOAc -pH 5.0
2-APB
200 pA
20 s
Control
10 mM HOAc - pH 5.0
300 pA
10 s
rTRPV2-D570N
rTRPV2-D570A
30 mM HOAc pH 5.0
750 pA
50 s
1 nA
1.5 nA
30 s
10s
1mM 2-APB
30mM HOAC pH5.0
30mM HOAC pH 5.0
Current Density [pA/pF]

Figure EV3. The role of pore residues for weak acid-sensitivity of TRPV2.

(**A**) Box diagrams with dot plots displaying the relative magnitudes of inward currents induced by 30 mM HOAc at pH 5.0 normalized with the responses evoked by 1 mM 2-APB in the corresponding cells ($n = 5$–10 for each mutant). (**B**) Box diagrams with dot plots displaying the current densities of heat-evoked currents provoked by 10 mM HOAc at pH 5.0 ($n = 6$–7 for each mutant). (**C**) Bar diagrams displaying mean (± SEM) current densities for currents evoked by 30 mM HOAc at pH 5.0 or 1 mM 2-APB ($n = 5$–8 for each mutant). (**A–C**) Data are demonstrated for rTRPV2-WT, -E561Q, -K566Q, -E569Q, -D570N, -E577Q, -E584Q, -E585Q, -E586Q, -D595N, -E599Q, -K602Q, -E609Q and −614Q. (**D**) Example of a current trace displaying that 30 mM HOAc at pH 5.0 fails to activate rTRPV2-K602Q. Note that the inward current evoked by 1 mM 2-APB is relatively small. (**E**) Heat-evoked currents in a cell expressing rTRPV2-K602Q. Note that 10 mM HOAc at pH 5.0 only provokes a minimal heat-evoked current. (**F**) Box diagrams with dot plots displaying the relative magnitudes of HOAc-evoked inward currents normalized with the responses evoked by 1 mM 2-APB ($n = 6$–7 for each mutant). Data are displayed for rTRPV2-WT, -K566Q, -K566A, -E569Q, -E569A, -D570N, -D570A -E577Q, -E577A, -E586Q and -E586A. (**G**, **H**) Representative current traces from HEK293T cells expressing rTRPV2-D570N (**G**) and rTRPV2-D570A (**H**). 30 mM HOAc at pH 5.0 evoked large currents in cells expressing rTRPV2-D570A. Functionality and expression was validated by application of 1 mM 2-APB. (**I**) Box diagrams with dot plots displaying the current densities of heat-evoked currents provoked by 10 mM HOAc at pH 5.0 in cells expressing rTRPV2-WT, -K566Q, -K566A, -E569Q, -E569A, -D570N, -D570A -E577Q, -E577A, -E586Q and -E586A ($n = 6$–7 for each mutant). (**J**) Bar diagrams displaying mean (± SEM) current densities for currents evoked by 30 mM HOAc at pH 5.0 or 1 mM 2-APB in cells expressing rTRPV2-WT, -K566Q, -K566A, -E569Q, -E569A, -D570N, -D570A -E577Q, -E577A, -E586Q and -E586A ($n = 6$–7 for each mutant). (**A**, **B**, **F**, **I**) The box denotes the 50th percentile (median) as well as the 25th and 75th percentile. The whiskers mark the 5th and 95 percentiles. Data points beyond the whiskers are outliers. Source data are available online for this figure.

© The Author(s)

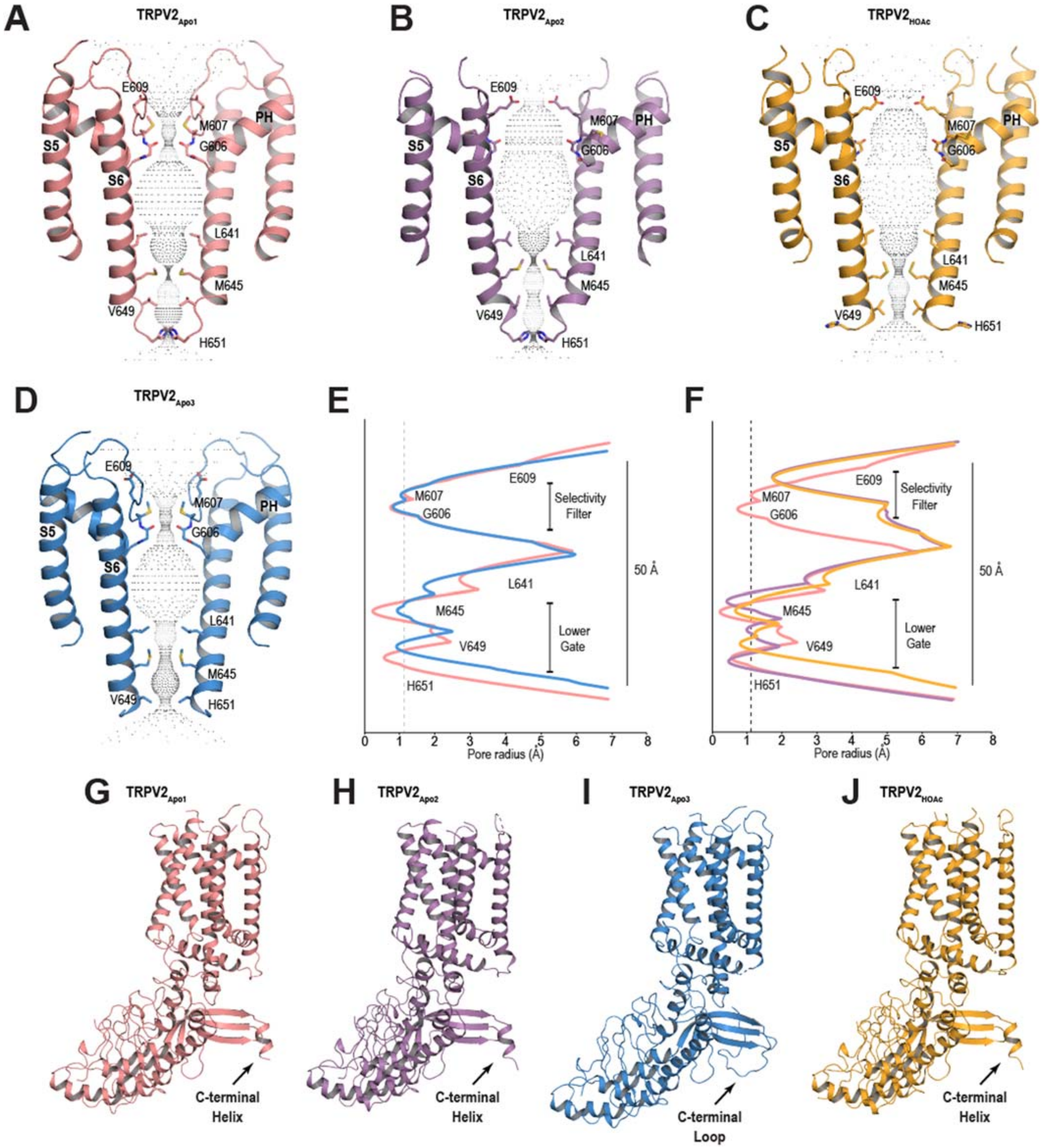

Figure EV4. The effect of HOAc on the pore profile of rTRPV2.

(**A–D**) Pore profiles of $TRPV2_{Apo1}$ (**A**), $TRPV2_{Apo2}$ (**B**), $TRPV2_{HOAc}$ (**C**), and $TRPV2_{Apo3}$ (**D**). (**E**, **F**) A graphical representation comparing the pore diameter of $TRPV2_{Apo3}$ (blue) to $TRPV2_{Apo1}$ (salmon) (**E**) or $TRPV2_{HOAc}$ (orange) to $TRPV2_{Apo2}$ (purple) and $TRPV2_{Apo1}$ (salmon) (**F**). The dotted line marks the radius of a dehydrated calcium ion. (**G–J**) Side view of a single monomer of $TRPV2_{Apo1}$ (**G**), $TRPV2_{Apo2}$ (**H**), $TRPV2_{Apo3}$ (**I**), and $TRPV2_{HOAc}$ (**J**). Arrows indicate C-terminus.

© The Author(s)

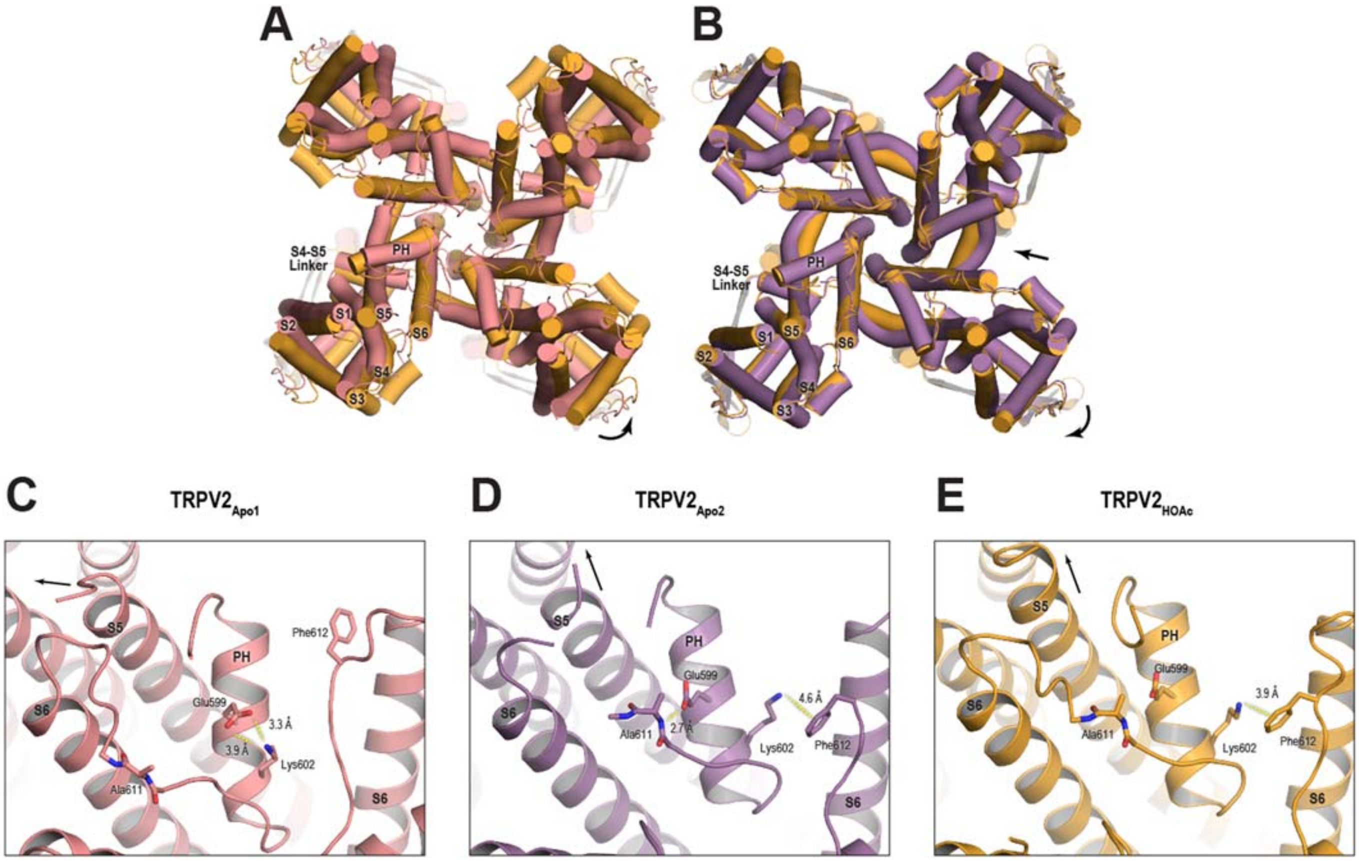

Figure EV5. HOAc-induced movements of rTRPV2.

(**A**, **B**). Alignments of $TRPV2_{Apo1}$ (salmon) vs. $TRPV2_{HOAc}$ (orange) (**A**) or $TRPV2_{Apo2}$ (purple) vs. $TRPV2_{HOAc}$ (orange) (**B**) at the extracellular face of the channel. Arrows indicate directions of movement in the transition from the apo states to $TRPV2_{HOAc}$. (**C–E**) View the pore helix in $TRPV2_{Apo1}$ (**C**), $TRPV2_{Apo2}$ (**D**), and $TRPV2_{HOAc}$ (**E**). Bonds between residues are indicated by a yellow dashed line. Arrows indicate trajectory of the top of S5.
